# Gleanings from Our Exchanges

**Published:** 1872-12-01

**Authors:** 


### Gleanings from Our Exchanges.

### HYPODERMIC MEDICATION.

We copy the following regarding Hypodermic Medication from the *Canada Lancet:*

We are indebted to Dr. Alexander Wood, of Edinburgh, for the discovery and application of Hypodermic Medication. It was first used by him in 1843, in the treatment of a case of neuralgia, and for many years its use was confined to the treatment of this affection, and morphine was the only agent so used. Wood believed that the remedy to be effectual should be localized, although he was well aware of its general effects on the system. Charles Hunter, of London, wrote an essay in 1859 on 'the Hypodermic treatment of disease,' in which he showed that localization of the injection was not necessary. He was an enthusiastic advocate of this plan of medication. From this time its use became very general throughout England and on the continent.

It was first used in America by the late Geo. T. Elliott of Bellevue Hospital, in a case of sciatica. Since then it has been gradually growing into favor among the profession, and is now very extensively used. But notwithstanding this rapid advance and its many advantages over ordinary medication, there are still many practitioners who have never tried it, and who do not think it possesses any advantages over the old way of giving medicine; some are prejudiced against it, and others regard it as an innovation or a novelty which is destined soon to be among the things that were. It has, however, in spite of all opposition assumed a wide range of application, both in the variety of diseased conditions to which it is applicable and the remedies used, and has taken its place as a standard means of great value to both the patient and practitioner in the relief of many painful and spasmodic diseases.

Remedies injected into the sub-cutaneous areolar tissue, have in most instances the same effect as when administered by the mouth. Some years ago a scientific committee was appointed by the chemical society of London, to report on the physiological and therapeutical effect of remedies administered sub-cutaneously, and they gave it as their opinion that no difference was observed in the effects of a remedy thus given, and by the stomach, except greater rapidity, certainty, and intensity of effect, and requiring a less amount to affect the system than when given in the ordinary way.

The agents thus used, being generally powerful in their nature, its application is not always unattended with danger, and therefore it is necessary to exercise care in its administration. Very great improvement has been made in the *instruments* now in use, and therefore nothing need be said regarding them, further than that those with a graduated glass barrel are preferable, as it enables one to see the quantity used, and also to be sure that no air occupies the barrel. One of the greatest dangers of this method, except its use in cardiac disease, is the risk of injecting air or the solution into a vein. This may always be avoided by pushing the needle through the integument (which has been pinched up for that purpose on the breast, arm, or shoulder) to the extent of three-fourths of an inch, and then withdrawing the point a short distance before injecting the solution. If air is drawn into the syringe in filling it, the instrument should be inverted, and the piston pushed in, till all the air is forced out.

Much of the success of this method of medication depends upon the purity of the medicines used, and the character of the solutions. The remedy should be in a perfect state of solution, and always filtered to remove any undissolved portions, as they are apt to give rise to the formation of small abscesses. The solution should not be too strongly acid or alkaline, and not too much concentrated. Pure distilled water only should be used, as a solvent, when practicable, and the solution should not be kept too long. We give below some of the formulæ in common use.

For Morphine: Magendie's solution is the best. It consists of Morphiæ sulph. grs. xvj; aquæ dest. ℥ j. Mix and filter. The dose is from 5 to 8 minims.

For atropine: ℞. Atropiæ sulph. gr. ss.; aquæ dest. ℨ iij. Mix and filter. The average dose is 4 minims. If it is desired to combine these two remedies, one grain of atropine may be added to Magendie's solution; of this 5 minims is the average dose.

For Strychnine: ℞. Strychniæ sulph. gr. j; aquæ dest. ℨ iij; acidi hydrochlor, gr. j. Mix and filter. Average dose 5 minims. It would be well to begin with a small dose and gradually increase.

For Quinine: ℞. Quiniæ sulph. grs. xx; acidi sulph. aromat, ten minims; aquæ dest. ℨ iij. Mix and filter. Nine minims equal one grain. This solution is more apt to cause abscess than the above, on account of its greater acidity.

For Calabar bean: ℞. Ext. calabar bean gr. ij.; aquæ dest. ℨ j. Mix and filter. The average dose of this is 8 minims.

For Corrosive sublimate; ℞. Hydrarg bichlor gr. j.; Aquæ dest. ℨ ij. Mix. Dose about 10 minims, and may be used every alternate day. It has been highly spoken of in the treatment of constitutional syphilis.

---

On Measuring the Chest. — Dr. Frœhlich, of Dresden, in a recent number of *Virchow's Archiv.*, gives for chest measurements the following simple directions, attention to which would insure uniformity of procedure: The person to be examined should stand in an unconstrained position before the physician, breathing with his mouth shut, and should raise both arms, stretching them out horizontally. A tape not broader than 1 Cm. (about ⅜ of an inch) should be placed round the chest directly under the inferior angles of the scapulæ behind, and the nipple in front, and should then be read off, first after the deepest inspiration and then after the deepest expiration, and both data recorded. The author then sums up the results which he has obtained by this method of observation, of which some of the more important are as follows: — The average circumference of the chest measured in 725 healthy men, 20 years of age, was, after deepest inspiration, 89 Cm. (about 35 inches), and after deepest expiration, 82 Cm. (about 32¼ inches), the average play of the chest being thus 7 Cm. A circumference of only 75 Cm. (29½ inches) indicates what the author calls an unripe chest, and should exclude the person from military service. A circumference of 750–759 Mm. should, under exceptional circumstances, be considered sufficient for military service; but when it reaches 760 Mm. (30 inches), if the person is otherwise healthy, then it ought to suffice.—*Med. Reporter.*

Pruritus of the Meatus.—Dr. Gruber (*All. Wien. Med. Zeitung*) states that the intolerable itching of the external auditory passage, which occurs in paroxysms, often periodical, takes place when uncomplicated by eczema, in persons past the middle period of life, especially those laboring under some disturbance of the circulation. For the radical cure, a solution of nitrate of silver, eight grains to the ounce of distilled water, is to be penciled on the part till the itching no longer occurs.—*The Doctor.*

Dr. Ira Remsen, lately instructor in the University Laboratory of Tubingen, has accepted the Chair of Chemistry and Physics in Williams College, Massachusetts.—*Med. and Surg. Reporter.*

